# Sol–Gel Synthesis of TiO_2_ with Pectin and Their Efficiency in Solar Cells Sensitized by Quantum Dots

**DOI:** 10.3390/gels10070470

**Published:** 2024-07-17

**Authors:** Jean Flores-Gómez, Silvia Mota-Macías, Juan P. Guerrero-Jiménez, Victor Hugo Romero-Arellano, Juan Morales-Rivera

**Affiliations:** 1 School of Engineering and Technological Innovation, University of Guadalajara, Campus Tonalá, Av. Nuevo Periférico No. 555, Tonalá 45425, Jalisco, Mexico; jean.flores9810@academicos.udg.mx (J.F.-G.); hugo.romero@academicos.udg.mx (V.H.R.-A.); 2 School of Science and Technology, University of Guadalajara, Campus Colotlán, Carretera Federal No. 23, Km. 191, Colotlán 46200, Jalisco, Mexico; silvia.mota@academicos.udg.mx

**Keywords:** TiO_2_ nanoporous electrode, dye-sensitized solar cell, pectin, synthesis, quantum dots

## Abstract

In this study, titanium oxide TiO_2_ nanoparticles were produced using the sol–gel approach of green synthesis with pectin as the reducing agent. The synthetized TiO_2_ nanoparticles with pectin were characterized by scanning electron microscopy (SEM), X-ray diffraction (XRD), visible light absorption (UV–Vis) and the BET method. The structure and morphology of the TiO_2_ powder were described with SEM, revealing uniform monodisperse grains with a distribution of 80% regarding sizes < 250 nm; the resulting crystal phase of synthetized TiO_2_ was identified as an anatase and rutile phase with a crystallinity size estimated between 27 and 40 nm. Also, the surface area was determined by nitrogen adsorption–desorption using the Brown–Emmet–Teller method, with a surface area calculated as 19.56 m^2^/g, typical of an IV type isotherm, indicating mesoporous NPs. UV–Vis spectra showed that sol–gel synthesis reduced the band gap from the 3.2 eV common value to 2.22 eV after estimating the optical band gap energy using the adsorption coefficient; this translates to a possible extended photo response to the visible region, improving photoactivity. In addition, the power conversion of the photoelectrode was compared based on similar assembly techniques of TiO_2_ electrode deposition. Quantum dot crystals were deposited ionically on the electrode surface, as two different paste formulations based on a pectin emulsifier were studied for layer deposition. The results confirm that the TiO_2_ paste with TiO_2_-synthesized powder maintained good connections between the nanocrystalline mesoporous grains and the deposited layers, with an efficiency of 1.23% with the transparent paste and 2.27% with the opaque paste. These results suggest that pectin could be used as a low-cost, functional sol–gel catalysis agent for the synthesis of controlled NPs of metal oxide. It demonstrates interesting optical properties, such as an increase in photo response, suggesting further applications to photocatalysts and biomedical features.

## 1. Introduction

Given the large and growing energy demand worldwide, alternative energy sources are being sought. One of these sources is solar energy because it is natural and renewable [1]. In the north of Mexico City, global horizontal solar radiation from 14 consecutive years was measured at 18.01 MJ/m^2^ day, which is equivalent to about 5.0 kWh/m^2^ [2]. This quantity indicates the large amount of possible energy that reaches this surface without being used. However, photovoltaic systems (PVSs) in urban areas are uncommon. [2]. PVSs represent a cost-effective and eco-friendly alternative to electricity users to decrease energy costs and reduce the amount of electricity that is drawn from the grid [3]. With increasing growth worldwide, PVSs are projected to reach 4500 GW by 2050 [4]. Historically, this technology has been divided into three generations, the first being silicon cells, developed in 1954, which currently generate efficiencies of 20% with monocrystalline silicon [5].

The next generation of heterojunctions or multi-junctions reaches efficiencies of 16% [6,7,8], and the third generation is dye-sensitized solar cells (DSSCs), an electrochemical cell based on the union of a semiconductor with a liquid electrolyte using a ruthenium complex [9]. These currently manage up to 12.5% efficiency in standard conditions and are currently a potential competitor [10] in simplifying the manufacture of solar cells. At the electrolyte interface, a reduction and oxidation process occurs where electron donors jump bands, leaving several gaps by favoring the injection of electrons into the conduction band and generating reference electron–hole pairs [11,12]. This electron transfer occurs vector-wise, which benefits the cell’s lifespan [13] because these electrons are absorbed on a nanoscale; therefore, a broader spectrum is excited during the interphase [11], and a nanostructured material is required since it takes advantage of the quantum confinement effect and charge transfer [14] movement between the semiconductor layers. These photoelectrodes or anodes are typically made of nanostructured materials such as titanium oxide, a material abundant on Earth, which is interesting since it is a semiconductor with a wide range of interesting and applicable properties, such as electrical, magnetic, optics, and catalytic properties, caused by different electronic structures in its oxidation states, its symmetrical magnetic behavior, and its acid–base properties [6,15]. It also has high corrosion resistance and a low biological reaction [10,16,17]. This kind of electrode is also a target of interest in most recent research involving photocatalysis, electrochemistry, and remediation. Naturally, TiO_2_ presents photoactivity in the UV region from 3.0 to 3.3 eV [18] depending on the phase structure. Only pure structures of anatase (space group I41/amd) or a mixture with rutile (space group P42/mnm) [19] are catalytic; otherwise, it needs to be doped or used in conjunction with a complex to increase adsorption. For the replacement of organic dye molecules [20,21] due to their low efficiencies [22,23] or the high costs of chemicals such as ruthenium [10,24], a cost-effective alternative is sensitizing it with quantum dots (QDs). Some of these metal chalcogenide QDs are CdS (cadmium sulfide), CdSe (cadmium selenium), PbS (lead selenium), InAs (indium arsenide), etc. [25,26]. Another approach to improve light harvesting and energy conversion is the manipulation of nanostructured materials [27]; furthermore, these electrodes can be manufactured without using high-tech equipment and with low-cost materials, following the tendency of finding sustainable ways of advancing this technology. Metal oxides synthesized with plant extracts should be further investigated as metal NPs [28]. Although the approach is similar, some reports such as Rajendhiran et al. suggest using prickly pear (opuntia) extract as a reducing agent with a predominate size of ~300 nm [22]; Fall et al. report the use of natural extracts of citrus sinensis with a 3–96 nm size range of the anisotropic material [18]; the group of Miu et al. synthetized TiO_2_ on *Camellia Shensis*, addressing the difficulty of obtaining reproducible green TiO_2_ NPs due the variability in the green extract, as apparently, the phytocompound is negatively influenced in the formation of a crystalline structure [29]. Kashale et al. report TiO_2_ NPs in a ~14 nm size range made of Bengal gram bean extract [30]. In reports on the use of pectin [31], it is commonly mentioned that this macromolecule composed of arabinan and galactic side chains can reduce metallics due to its aldehyde groups [28,31].

A typical photoelectrode is composed of (i) conductive glass, (ii) nanostructured semiconductor layers, (iii) polysulfide electrolytes, and (iv) a contra electrode [10,24]. The use of paste for screen printing is the easiest way to achieve semiconductor deposition; this deposit corresponds to three layers of TiO_2_: a first layer of compact TiO_2_, a second layer of transparent TiO_2_ paste, and a third layer of opaque (scattering layer) TiO_2_ paste.

The standard components of TiO_2_ paste are TiO_2_ nanoparticles, a solvent (e.g., terpineol), and a binder (e.g., ethyl cellulose) [7,32,33,34,35,36,37,38]. It is believed that this arrangement prevents short circuits during the transport of charge through metallic counter electrodes. It has also been indicated that this determines a cell’s electronic properties [32]. A cell’s interface with electrolytes influences the way energy levels are reduced; an electron infiltrates its mesoporous network, effectively reaching the conductive glass and, thus, moving. This results in a circuit with a counter electrode. A quantum dot is readjusted and restored while the hole is filled with an electron when it is reduced by that electron [5]. The adhesion of paste to conductive glass influences the success of charge generation and transport. Photoelectrode materials must also be homogeneous and nanostructured, as they must have a greater surface area with larger spaces for crystals with quantum dots to be deposited [12,39,40]. The method used for this deposition is called the successive ionic layer adsorption and reaction (SILAR) method [41], which is a simple and scalable method for the in situ growth of quantum dot nanocrystals [7]. In this work, we propose pectin as a replacement for the acid catalyst on an environmentally benign synthetic route with a low cost for metal oxide nanoparticles. A semiconductor paste for photoelectrodes in the semiconductor deposition layers was prepared using the doctor blade technique, and the SILAR method was used to sensitize the photoelectrodes. Then, we assembled a photosensitive cell, and the power conversion efficiency was measured with a solar simulator to obtain the Jsc–Voc curves. The nanostructured TiO_2_ material was characterized via XRD, UV–Vis, BET, and SEM to assess the morphology, phase crystallinity, surface area, and adsorption coefficient.

## 2. Results and Discussion

### 2.1. Characterization of the Sol–Gel Synthesis of TiO_2_ with Pectin

**Figure 1:**
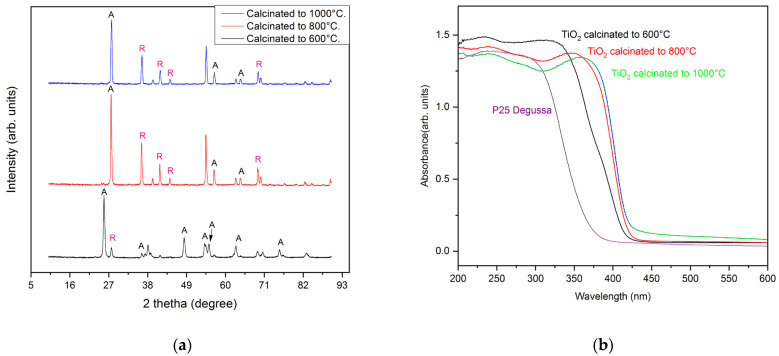
Characterization of the sol–gel synthesis of TiO_2_ with pectin at different calcination temperatures of 600 °C, 800 °C, and 1000 °C being A anatase R Rutile phase: (**a**) comparison of the XRD spectra of the crystalline phase of the TiO_2_ materials obtained at different calcination temperatures; (**b**) UV–Vis spectra of TiO_2_ materials compared with P25 Degussa.

We compared the materials according to the angles identified by the authors of [23,42,43] via X-ray diffraction, confirming the anatase phase at a distinct peak (2θ) at 25°, 37°, 48°, 54°, 55°, 62°, 71°, and 75°, which corresponded to diffraction from crystal planes (101), (103), (200), (105), (211), (204), (220), and (215). A rutile phase was confirmed at 27°, 36°, 41°, 49°, and 57°, which corresponded to diffraction from crystal planes (110), (101), (111), (210), and (220). These results suggest that the reduction with pectin stabilized the structure of the titanium oxide in its anatase and rutile phases, indicating that the calcination temperature influenced the crystallinity of the phase fraction. Figure 1a shows the synthesis of TiO_2_ in pectin calcined at different temperatures. The fitting of the results of the analysis revealed the phase crystallinity of the synthesized NPs. For pectin at 600 °C (bottom black graph), the crystallinity of the material was 73.5%, and according to the estimation, the size of the particles was around 27 nm. However, with the same method at 800 °C, the crystallinity of the material was 74.76%; according to the estimation, the size of the particles was around 40 nm. At 1000 °C, there was 64.30% crystallinity, and the size of the particles was 29 nm. This showed that there was an increase in the rutile phase in accordance with calcination temperatures greater than 700 °C. Similar results were reported by Fall et al. [44], who found an increase in the rutile phase after a pure anatase experimental result with calcination temperatures of >700 °C [18], implying that drastic sintering of the material could increase abrupt crystal growth. Additionally, the synthesized TiO_2_ presented central stability in the subsequent sintering process because of the phase purity [45]. Another consideration was the estimation of the average size of the crystallite using de Scherrer’s formula; the TiO_2_ varied from approximately 27 to 40 nm depending on the calcination temperature. The calculated values agreed with the values reported by other authors [46]. TiO_2_ demonstrated absorbance at the non-visible light level of 380 nm, which was in the UV region, unlike the absorption of the TiO_2_ synthesized with pectin as a reducer. Kashale et al. suggested that pectin remains present with titanium oxide as a composite, helping maintain a uniform size distribution even after calcination at 500 °C [30]. Figure 1b shows a notable difference, evidencing a slight redshift at almost 404 nm, which was closer to the visible region, covering a range from 380 to 400 nm. This behavior was present in all sol–gel syntheses of TiO_2_ with pectin at different calcination temperatures [47,48]. This alteration of the adsorption coefficient and photoactivity response of green synthetized TiO_2_ NPs was also reported in other works, such as [22,29]. It was concluded in both cases that a defined crystalline structure was not generated with the green synthesis. The development of defined nanostructured crystal phases ensures appropriate chemical functionalities at the surface, with anatase being more stable at the nanoscale [23].

**Figure 2:**
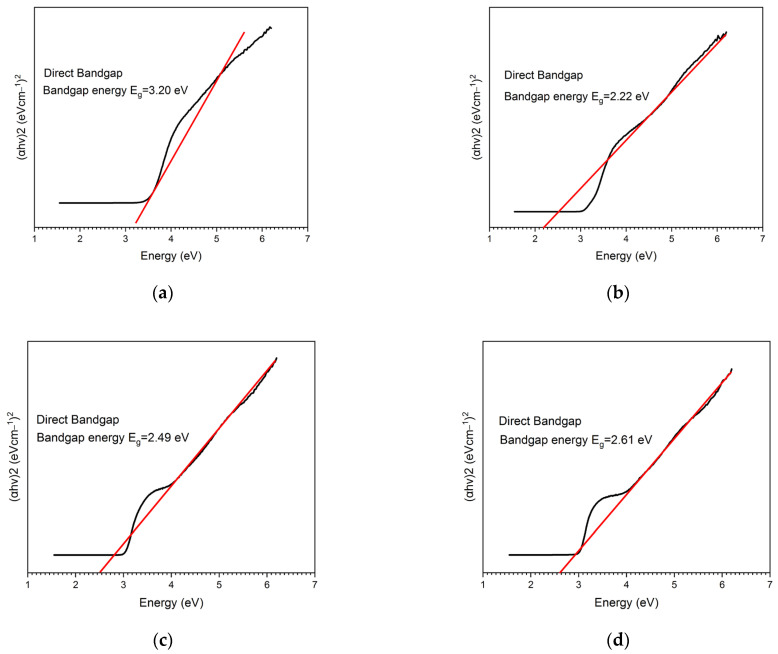
Tauc plots of P25 Degussa and the sol–gel synthesis of TiO_2_ with pectin at different calcination temperatures of 600 °C, 800 °C, and 1000 °C, being Red linear fitting, Black experimental data: (**a**) value of the bandgap energy estimated via the linear fit of TiO_2_ P25 Degussa (3.20 eV) in comparison with standard bandgap materials [18,28]; (**b**) bandgap energy estimated at 600 °C (2.22 eV); (**c**) bandgap energy estimated at 800 °C (2.49 eV); (**d**) bandgap energy estimated at 1000 °C (2.61 eV).

Optical adsorption measurements provide an accurate estimation of the optical band gap; Alsaal et al. mentioned that the wavelength has a coefficient spectrum associated with the energetic regions excited in lower and higher states, so an increase in the value of the adsorption coefficient probably occurs when there is a decrease in band transitions [49]. As Juvu et al. [50] discussed, this calculation is an estimation because semiconductors usually possess band transitions, so a direct application of Tauc can lead to erroneous estimates of the band gap. It is probably more accurate to take measurements below the scale if this starts from negative values to achieve a slightly more correct bandgap estimation. As observed in Figure 2, the bandgap was theoretically calculated with a linear fit of the Tauc plot for the sol–gel synthesis of TiO_2_ with pectin at 600 °C (2.22 eV), 800 °C (2.49 eV), and 1000 °C (2.61 eV) and for P25 Degussa with 3.20 eV. This difference in the band gap between the green-synthesized TiO_2_ NPs was also found in previous reports with 2.91 eV [22] and 2.71 eV [29].

**Figure 3:**
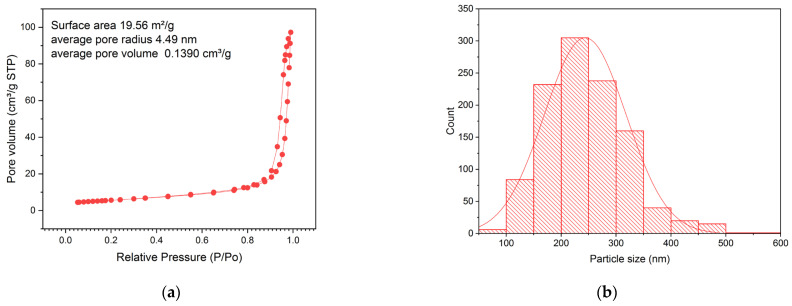
Surface area and NP size distribution: (**a**) N2 adsorption–desorption isotherm of the sol–gel synthesis of TiO_2_ with pectin to evaluate the surface area; (**b**) bar chart histogram indicating the average size of TiO_2_ NPs obtained through the sol–gel synthesis of TiO_2_ with pectin.

The surface area was determined via nitrogen adsorption/desorption using the Brunauer–Emmett–Teller (BET) method [51]. The type IV isotherm was displayed with a pressure (P/Po) ranging from 0.5 to 0.9, as observed in Figure 3a. Typically, a hysteresis loop with this adjustment indicates a mesoporous material, which is attributed to the release of carbon dioxide during the calcination of organic biomolecules that disperse a metal oxide [22,30], thus causing the remaining alkoxyl groups of the pectin to have a uniform size distribution and surface area. According to surface theory, this is divided into an external surface, which is defined as the surface outside the pores, and an internal surface, which is the surface of all of the pore walls [51]. The calculated surface area was 19.56 m^2^/g, with an average pore volume of 0.13904 cm^3^/g and a pore radius of 4.49 nm. These results are consistent with what was reported by [30]: 4.29 nm and 0.2372 cm^3^/g, respectively. Because the pores had widths greater than 2 nm, the material was defined as mesoporous [51]. In Figure 3b, the distribution count of the sol–gel synthesis of TiO_2_ with pectin NPs can be observed; most of these were below 300 nm, and 80% of the distribution contained sizes within the range of 120 to 248 nm, which was optimal as an opaque mesoporous paste [33,34].

**Figure 4:**
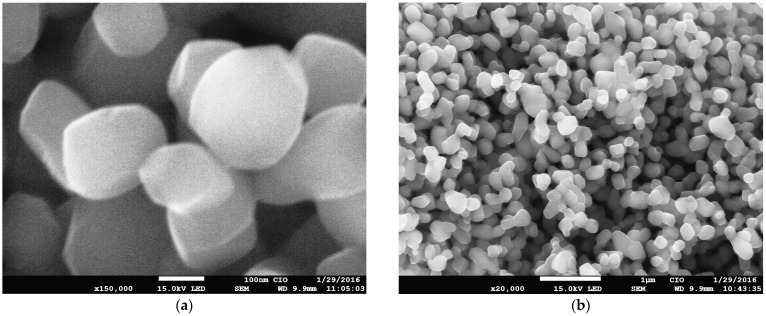
SEM micrograph of the sol–gel synthesis of TiO_2_ with pectin: (**a**) closeup with a distinguishable grain shape; (**b**) micrograph of the uniformity of the size of the TiO_2_ nanoparticles.

It was verified that the morphological structure of the nanostructured mesoporous material obtained from the sol–gel synthesis of TiO_2_ with pectin was similar to that obtained through traditional hydrothermal synthesis. Figure 4a confirms that the TiO_2_ NPs had a uniform size distribution in small aggregate groups even after calcination; this was a result of the use of pectin as a reducing agent for the synthesis of uniform titanium dioxides. Although the NPs formed were not complete nanospheres, they were conveniently structured and dispersed, and presented a crystalline phase, unlike in previous reports, which indicated that plant extracts such as epigallocatechin-3-gallate (EGCG) from green tea (*Camellia sinensis*) did not influence the crystalline phase in TiO_2_ synthesis [29], and reported that the rate of reduction in extract concentration was influenced by the TiO_2_ precursor and influenced the size of NPs [22], as well as demonstrating that anisotropy and aggregations of NPs were detected although there were crystalline phases [18]. In Figure 4b, the sizes are easily distinguishable, and the particles are uniform. It was mentioned in other reports that the synthesized materials created an efficient way to shorten diffusion paths and improve charge transport in interphase cells in contrast to commercial products [45]. Furthermore, the size and shape of the nanospheres commonly found in reports on green synthesis [28] showed greater stability when generated in sol–gel media [12,16,30]. As sol–gel synthesis is a simple method for minimizing the complexity of the hydrolysis of precursors and for the stabilization of the tuning morphology, this synthetic route is also a good replacement for hazardous reagents using acidic catalysts, as it provides a low environmental impact through the preparation and application of TiO_2_, since the hydrothermal method continues to be the current trend for the preparation of nanostructured metal oxide phases [23]. Additionally, it has been reported that adding organic chains [22,28,30] such as pectin could increase the porous surface, thus providing viability to the process of obtaining an anatase mesoporous phase, which is desirable as a crystalline phase with sizes greater than 100 nm. This is ideal for an opaque scattering paste [52], and highly porous electron collectors of QD crystals can be attached to sensitize photoelectrodes [53,54].

**Figure 5:**
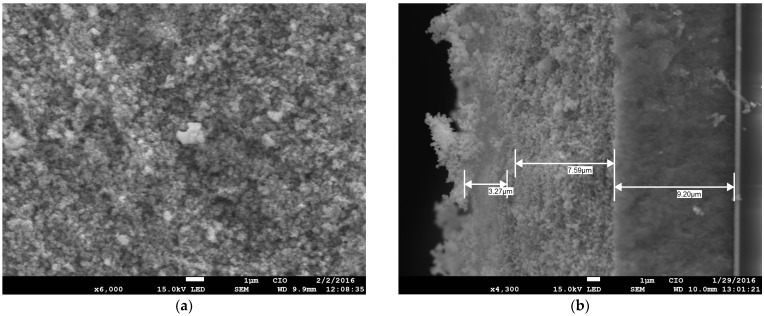
SEM micrograph of the TiO_2_ layers; (**a**) closeup from above, seemingly without defects; (**b**) micrograph of TiO_2_ layers with thickness measurements.

We mentioned before that the paste deposit corresponded to three TiO_2_ layers: a first layer of compact TiO_2_; a second layer of transparent TiO_2_ paste (initially, this layer needs to be approximately 10–14 μm); and a third layer of opaque (scattering layer) TiO_2_ paste with a thickness of 4–5 μm and ideal anatase-phase particles [34,35]. This is because the most stable crystalline phase for chemical functionalities on the interface of solid-state diffusion through photoelectrode layers is anatase. The influence of the thickness of the semiconductor layers is attributed to the fill factor (FF%) and hole transport, which translates into efficiency [49]. It has been reported that the optimal thickness is three layers of semiconductor material [27,34,35,55]; below and above this number, the loss of injected electrons was detected during photoconversion. Figure 5a shows the surface of the electrode from above; in Figure 5b, a transverse section shows the arrangement of the layers and their average thicknesses measured using SEM. On the surface of the final opaque layer, there was no complete adhesion, demonstrating the difficulty of finding ideal viscosity conditions for the deposit, although this can also be attributed to the cutting process of the assembly photoelectrode with the films already deposited.

#### Characterization of the Performance of a DSSC Cell with the Pectin-Based Paste

**Figure 6:**
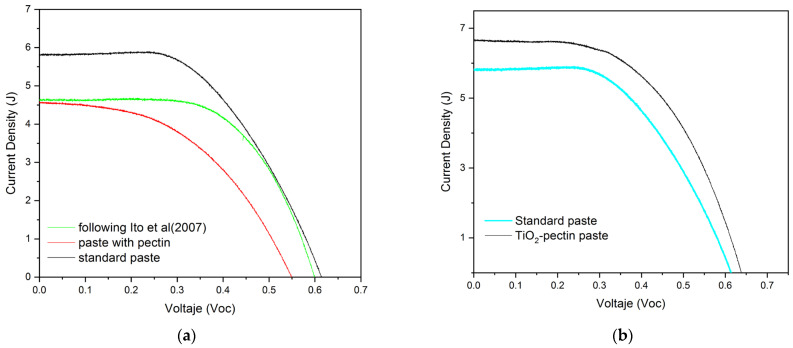
Jsc–Voc curves: (**a**) samples prepared with transparent P25 paste with a pectin agglutinant and QD sensitization, compared to Ito [31]; (**b**) samples prepared with an opaque paste through the sol–gel synthesis of TiO_2_ and a pectin emulsifier with QD sensitization.

In a simulator, the voltage difference between a working electrode and a reference electrode was measured. Five repetitions or measurements were carried out for each sample, and each measurement was compared with a blank (the standard reference paste formulation). Figure 6a shows that pectin was viable as a binder in place of ethylcellulose in the transparent paste formulation [34,35]. In fact, we believe that this is the first report of pectin as an emulsifier and that the apparent porosities generated by this chain within the paste after the sintering function efficiently generated and retained QDs and electrolyte diffusion. Additionally, Figure 6b confirms that the sol–gel synthesis of TiO_2_ with pectin generated a viable material with optical characteristics for the current generation of solar cells used as photoelectrodes. Other reports of TiO_2_ synthesized with green methods reported similar efficiency, with a maximum of Ƞ = 1.4 in organic dyes [22]. Traditional sol–gel photoelectrode fabrication with TiO_2_ using sprayed pyrolysis layers resulted in an efficiency of Ƞ = 2.44, and TiO_2_ made through hydrothermal synthesis had an efficiency of Ƞ = 3.94; these results are notable.

We explored the development of the photoconversion efficiency, as shown in Table 1, proving that it is possible to propose alternative eco-friendly synthetic routes and alternative components that could increase photoconversion efficiency [53] with the manipulation of the nanostructured material to improve light harvesting in electrode semiconductor devices [49]. We can also recall that photoelectrodes with multiple layers of TiO_2_ facilitate the percolation of charge in nanostructured networks due to the correct diffusion through the roughness and greater dispersion centers with electrolyte and QD nanocrystals [6,34]. This possibility indicates the conversion of more photons into current, as it covers most of the visible spectrum, leading to higher efficiency during device formation [22]. We can attribute this to a good contact surface ratio of the deposition paste.

## 3. Conclusions

With the proposed sol–gel synthesis of TiO_2_ with pectin as a reducing agent, NPs were obtained via a low-cost, environmentally friendly method that provides an alternative to hazardous reagents in traditional complex synthesis. XRD patterns confirmed that NPs were obtained in the anatase crystalline phase with a mix of the rutile phase at calcination temperatures greater than 700 °C; at 600 °C, the crystalline phase comprised 73.53%, with a mean size of 27 nm. The BET method indicated that this was a mesoporous material with a surface area calculated as 19.56 m^2^/g. NPs were obtained as the result of a uniform size stabilization promoted by diluted pectin as a reducing agent, which limited the size distribution and surface area according to the biomolecule chains present in the sol–gel synthesis. According to the adsorption coefficient, the direct band gap estimation of the synthesized NPs decreased from the standard value of 3.2 eV to 2.2 eV; this difference suggested an alteration in the photoactivity with extended photo-response conversion or increased light harvesting. We concluded that this displacement was beneficial to the cell’s optical properties and material performance. In combination with a paste formulation for TiO_2_ layers, we fabricated DSSC using the TiO_2_ synthesized with pectin. The last scattering layer had the main function of collecting sensitized QDs on a highly porous substrate to promote optimal hole–pair recombination on the photoelectrode interphase. Our QDSSC exhibited power conversion efficiency at (2.27) with an FF of 54.7%. These values correspond to the maximum efficiencies due to low series resistance. Additionally, when we formulated the paste, pectin was also used as a binder; its combination with TiO_2_ synthesized with pectin suggested that further interaction with the polymeric chain occurred during the reduction process and that oxygen dissolved during the proposed synthesis. This was essentially a positive result for the proposed green synthesis using pectin, as it promoted spaces between the TiO_2_ layers after sintering. These findings suggest that this is an innovative and reproducible approach to the synthesis of TiO_2_ with uniform morphology and size and with a narrow band gap and interesting photo-response, making it interesting for wider applications. Additionally, these photoelectrode layers need to be tested with other methods, such as dyes, to analyze the proposed synthesis of TiO_2_ as a semiconductor electrode. It is also essential for the efficiency that paste deposition is improved for both transparent and opaque pastes; thus, with improvements in the design of these materials, very promising results can be achieved.

## 4. Materials and Methods

### 4.1. Chemicals and Reagents

Titanium (IV) isopropoxide (97%), acetylacetone (>99%), sodium hydroxide (NaOH), pectin from citrus peels (74.0 (dried basis)), titanium (IV) oxide nanopowder, P25 with a primary particle size of 21 nm (TEM) (99.5% trace metal basis), α-Terpineol (96% mixture of isomers), cadmium acetate dehydrate (Cd(CH_3_COO)_2_.2H_2_O), sodium sulfide (Na_2_S 99%), zinc acetate dihydrate (Zn(CH_3_COO)_2_.2H_2_O), and ethyl cellulose (48% ethoxyl, viscosity 10 cP) were acquired from Sigma-Aldrich (Sofia, Bulgaria). Sodium sulfide (Na_2_S.9H_2_O) and sulfur (S) were acquired from KARAL. Fluorine-doped tin oxide (FTO) conductive glass was obtained from MTI (TEC-15). Distilled water (DW) and ethanol (E) were also used for dilution.

### 4.2. Sol–Gel Synthesis of TiO_2_ with Pectin

First, 2 g of pectin was placed in 20 mL of DW while stirring at 100 °C. In a beaker, 100 mL of DW was added to dissolve 11.5 mL of titanium (IV) isopropoxide. Afterward, the previously diluted pectin was added with a controlled drip for 10 min. It was stirred for 3 h, washed three times with E and once with DW in a centrifuge for 5 min at 3700 rpm, and dried at 80 °C for 24 h. This was followed by high-temperature treatments at 600 °C, 800 °C, and 1000 °C for 6 h, resulting in TiO_2_ NPs, which were collected and stored.

### 4.3. Formulation of the Pectin-Based Paste

The paste was formulated with our proposed modification of the method by Ito [33], which was adapted to our lab conditions. First, 1.5 g of TiO_2_ synthesized with pectin or P25 Degussa was added to a mortar; then, 1 mL of acetic acid was added and mixed for 5 min, 250 µL of DW was added 20 times, mixing for 1 min each time, and 750 µL of DW was added six times, mixing for 1 min each time. Then, 50 mL of DW was added to the mortar and mixed for 1 min. This mix was sonicated for 1 min, and 5.6 mL of alpha-terpineol (α-Terpineol) was added (to increase shelf life [27]) and sonicated for another 1 min. Then, 5 mL of pectin solution was added and stirred for 1 min (as a binder for tuning the paste viscosity and then porosity, leaving vacancies after sintering [27]), and a rotary evaporator was used for 1 h to obtain the TiO_2_ paste. The paste formulations followed the same route; only the TiO_2_ powder component was different in the transparent TiO_2_ paste (made with P25 with a typical particle size of 20 nm) and the scattering or opaque paste (made with the sol–gel synthesis of TiO_2_ with pectin NPs with a typical particle size of 200 nm).

### 4.4. Fabrication of Photoelectrode Using Pectin Synthesized TiO_2_ NPs

FTO conductive glasses were washed with isopropanol acetone and DW. Afterward, the first layer of compact TiO_2_ was deposited via spray pyrolysis over the FTO glass, and a solution of 9 g of titanium (IV) isopropoxide (at 0.2 M) with acetylacetone/ethanol (1:1) was used to create a size of 200 +/− 5 nm; this was followed by sintering for 30 min at 450 °C. Then, the doctor blade coating method was used for subsequent layers of the transparent (P25 TiO_2_) and opaque (pectin-synthesized TiO_2_ NPSs) paste to control the thickness at 6 mm, and Scotch tape was fixed on the FTO glasses. Finally, the layers were sintered for 30 min at 450 °C to achieve good electrical contact between the NPs and to evaporate the organic solvents [56,57].

#### 4.4.1. Sensitization with Quantum Dots

The SILAR method was used to sensitize the photoelectrode, and the CdS (core) and ZnS (shell) were deposited to enhance the photovoltaic performance; a single cycle consisted of 1 min of dip-coating in 0.05 M Cd ((CH_3_COO)_2_.2H_2_O) for the Cd^2+^ source and 0.05 M Na_2_S for the S^2−^ source. Both precursors were dissolved with ethanol and methanol/water (ratio: 1/4; 1:1). During the successive dipping steps, the electrodes were systematically cleaned by submerging them in the corresponding solvents (ethanol and methanol/water) to eliminate excess precursor content. The SILAR method was performed for seven cycles to obtain a constant photoelectrode coverage with CdS QDs. To reduce the recombination of electrons in the TiO_2_ layers with the polysulfide electrolytes [45,46] and to enhance the photovoltaic performance, a second SILAR procedure with 0.1 M Zn ((CH_3_COO)_2_.2H_2_O) for the Zn^2+^ source and 0.1 M Na_2_S for the S^2−^ source was deposited for a 1 min dip in two cycles. ZnS is a popular photoelectrode coating material for preventing interfacial recombination, as it is a semiconductor with a wide band gap of 3.6 eV [24,37].

#### 4.4.2. Fabrication of Counter Electrode

A counter electrode needs to meet some requirements, such as being highly electrocatalytic, catalyzing reduction on polysulfide species, being compatible, not dissolving in polysulfide electrolyte, undergoing photodecomposition, functioning in the range of 4.3–5.3 eV, being a good electron conductor, being inexpensive, and being nontoxic [24]. In terms of these parameters, the Cu_2_S counter electrodes yielded high performance with QDs only below the nanostructured fabrication of the carbon and platinum layer [9,24,34,35]. Cu_2_S counter electrodes were constructed by immersing brass foil in HCl solution (38%) for 1 h at 90 °C; then, they were immersed in polysulfide electrolyte for 1 min until the etched portion turned black, indicating the formation of cuprous sulfide. This is an established protocol for growing a Cu_2_S layer from a brass substrate while using low-cost components and a facile process.

### 4.5. Cell Configuration Assembly

**Figure 7:**
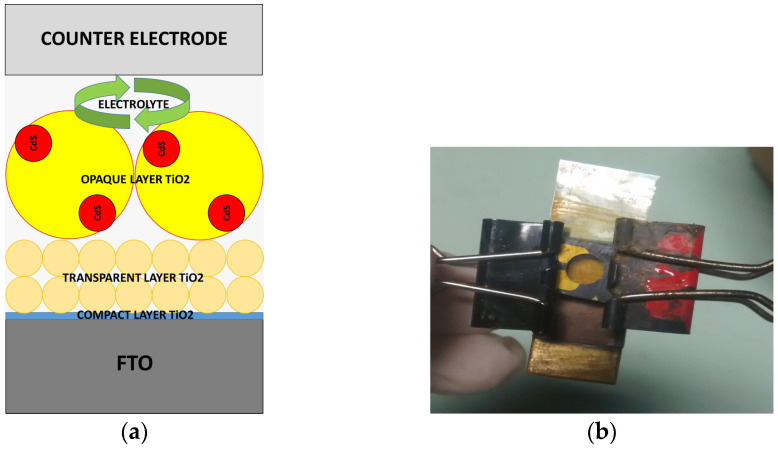
Cell configuration assembly. (**a**) Schematic representation of the assembly of solar cells; FTO, compact TiO_2_, transparent TiO_2_, opaque TiO_2_, Cd and Zn sulfide quantum dots, electrolyte, counter electrode. (**b**) Photo of the QDDSSC assembly from above.

To test the performance of the photoelectrode and to measure its photovoltaic efficiency, it was necessary to fabricate a sensitized solar cell with QDs by assembling sensitized TiO_2_ layers deposited on FTO glass and a Cu_2_S counter electrode and adding a 20 µL drop of polysulfide electrolyte using a Scotch space. Sensitized TiO_2_ layers with a QD photoelectrode (anode) and a Cu_2_S counter electrode (cathode) were kept opposite and bound using binder clips. A mixture of 1.0 M S, 0.1 M NaOH, and 1.0 M Na_2_S, which were diluted with DW, was used for the polysulfide electrolyte. This allowed the creation of an interphase between layers for diffusion prior to subsequently taking Jsc–Voc measurements with a solar simulator. Figure 7a shows a schematic representation of the assembly of solar cells, and Figure 7b shows a photo of the steps of the assembly’s configuration.

### 4.6. Characterization of the Sol–Gel Synthesis of TiO_2_ with Pectin

The surface morphology of the NPs was assessed using scanning electron microscopy (SEM). These images were obtained with a JEOL JSM-7800 microscope. Ultraviolet–visible (UV–Vis) absorption spectra were recorded using an Agilent Technologies Cary Series UV–visible–NIR spectrophotometer (Cary 5000), and the BET surface area was measured by Quantachrome Corp. The crystalline structure was described based on X-ray diffraction (XRD) patterns using a BrukerD-2 PHASER.

The crystallite size (D) was obtained using the Debye–Scherrer formula, which is described by Equation (1).

(1) $$D=\frac{k\lambda }{\beta \cos\theta }$$

where k = 0.9 is the constant of Scherrer for tetragonal TiO_2_, λ is the wavelength of the source, λ = 0.15418 nm, β is the full width at half maximum (FWHM), and θ is the Bragg angle [58].

Using the Tauc model, we plotted the estimates of the optical bandgap energy (*Eg*) from the UV–Vis spectra using Equation (2).

(2) $$\left(\alpha hv\right)=A\left(hv-Eg\right)n$$

where α is the absorption coefficient, A is a photonic-energy-independent constant, *h* is the Planck constant, *v* is the photon frequency, and *n* is a factor that depends on the electronic transition (1/2 when equal, 2 when direct or indirect) [22,50].

### 4.7. Characterization of a DSSC Cell with the Synthesis of TiO_2_

Current density–voltage (J–V) curves were evaluated with a 600 Gamry Potentiostat as a reference scanning from 0 to 600 mV at 100 mV/S. A solar simulator (Oriel Sol 3) was used to measure the current. The light intensity was adjusted by employing an NREL-calibrated Si solar cell with a KG-2 filter for one instance of sunlight intensity (100 mW/cm^2^). The experimental results were fitted with the Origin8 software. The photoconversion efficiency Ƞ and fill factor (FF) were calculated with Equations (3) and (4).

(3) $$FF\left(\%\right)=\frac{Pmax}{Jsc\;Vco}\times 100$$

(4) $$Ƞ\left(\%\right)=\frac{Pmax}{Pi}=FF\frac{Jsc\;Voc}{Pi}$$

where Ƞ (%) is the efficiency, the maximum electrical power from the incident solar power conversion in the cell; FF (%) is the filling factor, the maximum power obtainable from the deviation that the cell presents in the induced area; Jsc (mA/cm^2^) is the short-circuit current density; Voc (mV) is the open-circuit voltage; Pi (mW/cm^2^) is the incident light power density (100 mW/cm^2^); Pmax represents the relationship of the maximum obtainable electrical power.

The higher the fill factor (FF), the more electrical energy can be extracted, so that from the previous equations, we have the following: the results are influenced by many photo-electrode factors, such as the texture of the films, electron recombination, and the transmittance within the cell [43,53].

## Figures and Tables

**Table 1:** Photovoltaic response of differently structured photoelectrodes in TiO_2_ solar cells at an intensity of 100 mW/cm^2^.

Configuration	Voc (mV)	Jsc (mA/cm^2^)	FF (%)	Ƞ (%)	Reference
TiO_2_/CdS/Cu_2_S	8.3	474	41.3	1.56	[53]
TiO_2_/CdS/ZnS/Cu_2_S	7.2	504	52.9	1.94	[53]
TiO_2_/CdS/ZnS/ZrO_2_ with Yb3þ/Er3þ/Cu_2_S	7.1	593	68.7	3	[53]
TiO_2_/Bi2s3/CdS/Zn/Cu_2_S	9.3	—	53.7	2.52	[54]
TiO_2_/DB/N719/CC	10.96	—	55	3.81	[27]
FSPTiO_2_/DB/N719/PT	5.25	720	64	2.44	[55]
HTTiO_2_/DB/N719/PT	13.46	660	44	3.94	[55]
TiO_2_/COM/N719/PT	19.56	720	54	7.67	[55]
GSHTiO_2_/PPE/RB/CC	3.22	660	55	1.16	[22]
GSHTiO_2_/PPE/EY/CC	2.85	640	48	0.8	[22]
GSHTiO_2_/PPE/PP/CC	3.49	660	61	1.4	[22]
TiO_2_/DB/CdS/Cu_2_S	12.56	723	31.7	2.88	[22]
TiO_2_/CdS/ZnS/Cu_2_S	5.7	610	54.1	1.88	This article
TiO_2_/PNB/CdS/ZnS/Cu_2_S	4.5	560	48.9	1.23	This article
SGPNTiO_2_/PNB/CdS/ZnS/Cu_2_S	6.5	640	54.7	2.27	This article

Meanings of the acronyms: standard TiO_2_ (P25), CdS: sulfur cadmium QDs, COM: commercial Titania electrode (Solaronix S.A), FSP: spray pyrolysis synthesis of TiO_2_, ZnS: sulfur zinc QDs, DB: doctor blade deposition, HT: hydrothermal synthesis of TiO_2_, N719 dye, Cu_2_S: copper sulfide counter electrode, GSH: green synthesis, Bi2s3: bismuth sulfide QDs, PT: platinum counter electrode, SGPNTiO_2_: sol–gel synthesis of TiO_2_ with pectin, Yb3þ/Er3þ: o-doped ZrO_2_ nanoparticles, CC: carbon counter electrode, EY: eosin yellow synthetic dye, — not reported, PPE: prickly pear (opuntia) fruit extract, RB: rose Bengal synthetic dye, PNB: paste with pectin binder, PP: prickly pear (opuntia) dye.

## Data Availability

Data is available by corresponding author upon request.
